# Remarks on the Prunus Virginiana, Particularly the Wine and Ferrated Wine of Wild Cherry

**Published:** 1869-01-15

**Authors:** X. Toner

**Affiliations:** Albany, New York


### *Remarks on the Prunus Virginiana, Particularly the Wine and Ferrated Wine of Wild Cherry.*

BY X. TONER, M.D., ALBANY, NEW YORK.

The medicinal bark of the Prunus Virginiana is the inner bark of the Cerasus Serolina, or Wild Cherry, a large tree indigenous in the United States. The bark may be obtained from the root, trunk, or branches of the tree—that of the root is preferred by many. Deprived of its epidermis, it is of a reddish-yellow color, brittle, and easily pulverized. When fresh, it has the smell of peach leaves or bitter almonds. Its taste is bitter, astringent, and somewhat aromatic. Its active principles are tannic acid and amygdalin. In the process of distillation traces of hydrocyanic acid have been discovered.

The *physiological action* of the wild cherry is two-fold, viz: that of a stimulant or tonic to the digestive function, and, by reason of its amygdalin, as a direct sedative to the nervous system, and through that upon the circulation. *Dr. Eberle* states that under the influence of copious draughts of the infusion (℥ i—Oij), continuously kept up, the pulse fell from seventy-five to fifty per minute ; but immediately upon the suspension of the draughts of the infusion the pulse returns to its normal condition.

In regard to its *therapeutical* action there is very general agreement among all who have used it to any considerable extent. Few remedies, in fact, have had a more happy fortune in this respect. We quote, as expressing very happily the well-nigh unanimous opinion of the profession, from Professor Wood, in relation to the crude article or the domestic infusion :

" The joint tonic and sedative properties of this bark admirably adapt it to the treatment of cases of general debility, with enfeebled digestion, an irritable state of the nervous system, and excessive frequency of pulse. Long before its chemical peculiarities were discovered, experience had established this application of the remedy. In the treatment of *pulmonary consumption* it has for many years been a favorite in this country, and, before cod-liver oil came into notice, was probably more relied on than any other single medicine. It was employed not only in the advanced stages, when *hectic fever* had set in, but from the beginning, and often as a preventive, in cases where a strong tendency to the disease seemed to be displayed. It was given with a view of imparting tone to the digestive organs and system generally, and thereby modifying the tuberculous diathesis, and was preferred to other tonics because it was thought to produce these effects with less danger of undue excitement. Now that it is known to be positively sedative to the heart, and to the nervous system, we can better understand its usefulness in that complaint. In other forms of *scrofulous disease*, presenting a similar complication of debility of the digestive and nutritive functions, with frequency of the pulse, it is equally indicated. Few remedies are better adapted to *hectic fever*, from whatever source it may proceed. In the debility of *convalescence from fevers* and other acute diseases, when attended, as it often is, with night sweats, a frequent pulse, and sleeplessness, restlessness, or other functional nervous disorder, the wild cherry bark is also an excellent remedy."

The various preparations of wild cherry, in so far as they are efficacious, act by virtue of the same principle; but certain of them, as we shall presently see, have been so prepared as to unite the virtues of the wild cherry with the specific properties in a very useful manner. They are chiefly beneficial in debility, and convalescence from acute diseases, uniting, as they do, with a tonic power the property of quieting irritation and diminishing nervous excitability. Their use is indicated in all cases requiring a general tonic, particularly the impairment of the constitution by dyspepsia, debility resulting from inflammatory fevers, and in those cases where the digestive powers are impaired with general or local irritation existing at the same time. Also, owing to their slightly astringent properties, they are beneficial in the summer complaints.

They have not the power to interrupt the paroxysms of intermittent fever, but they can assist to keep off the paroxysms when once they are broken up, and when it is evidently injurious to continue the quinia.

In speaking of the remedies for chlorosis, Dr. J. Bates writes, concerning the excellent and delightful preparations of *Messrs. Tilden & Co.:*

"The *wine* of wild cherry is a very excellent preparation, and should be administered in connection with other remedies usually given in this malady. The wine, if scientifically prepared, will be found advantageous in this complaint, even when given alone. It may be used in doses of a dram to half a wine-glass three or four times a day. Undoubtedly the best preparation of this agent for the treatment of chlorosis is the *ferrated wine* of wild cherry. Diseases of this class, uncomplicated, will in most instances be signally relieved by this valuable combination. By uniting the hærmatinic and tonic properties of iron with the sedative and tonic properties of the prunus, in the ferrated wine of wild cherry, a happy union of medical properties is accomplished, which renders it more useful in a great variety of atonic diseases, and far more efficient than the same agents would be if administered separately."

But it is not alone in chlorosis that the value of these preparations we are speaking of is exhibited. In all diseases where there is a reduction of the red globules of the blood, especially in anæmia, the *ferrated wine of wild cherry* will be found to prove its success.

Dr. Flint, in speaking in reference to the therapeutics of the anæmia of *tuberculosis*, *Bright's disease*, *&c.*, writes:

"When it is the chief condition to be met therapeutically, the first points are to remove, if practicable, the cause or causes on which it depends. The next point is to employ measures to restore the normal quantity of red globules. * * * * *First*, a nutritious alimentation. * * * * *Second*, the use of tonics and stimulants. * * * * *Third*, iron as a special remedy. * * * * *Fourth*, a regimen calculated to increase the energy of the assimilative functions." * * * *

To meet the second and third points, there is no better tonic and stimulant preparation than the *ferrated wine* of wild cherry, given in doses from one to four drams three times a day.

This *ferrated wine* of wild cherry I regard as an *exceedingly valuable preparation*, since it so *happily* and *perfectly* unites the blood-restoring iron with general tonic and nervous sedative effects. We have prescribed it in a large number of cases where the nervous system was prostrated, and for the legionic symptoms concomitant of general debility, and have been extremely gratified by its prompt and efficient action. In fact, we have found no one of the general vaunted remedies *superior*, or even of *equal* efficiency to it to combat such morbid conditions.

The *ferrated wine of wild cherry*, as extemporized from the fluid extract, we have found from observation is *vastly inferior* to that scientifically prepared by the firm of Tilden & Co., both in its physical appearance and therapeutical action. Already have we seen it in market spuriously prepared, which is having its evil tendencies, and must inevitably result in throwing into disfavor the *honest article.* It is unfortunate that there is scarcely a medicinal preparation which professes any marked advantages, placed in the hands of the physician, before competition goes to work todestroy its efficacy by means too palpable to require enumeration here.

Dr. Allbut (in Braithwaite's Retrospect, part 55, page 258,) speaks very highly of the wild cherry. He says, "I have found the wild cherry useful not only in cases of cardiac disturbance, but also of general nervous excitability, of atonic dyspepsia, and of intestinal irritability. It seems, however, to have a more special bearing upon the arterial nervo-muscular tissues, as digitalis also has, and in proper cases it comes as a valuable substitute for digitalis when this medium is ill borne." Dr. A. declares that the bark of the wild cherry in doses under one dram of the tincture and under one ounce of the infusion does not nauseate, as do *opium*, *digitalis*, *conium*, and other sedatives, though, indeed, it will not take the place of digitalis in extreme cases."

Dr. Eberle, in speaking of the medicinal value of the wild cherry bark, says, "It lessens the frequency, tension, and irritated state of the pulse, moderates the cough and profuse nocturnal perspirations, checks the diarrhœa, and sustains the general strength of the system.

In *amenorrhœa* the combination of the ferrated wine of wild cherry and stramonium has proved valuable.

In *pertussis* the fluid extract of belladonna, with the syrup of wild cherry, has been serviceable in some cases.

After all, what we claim especially for the *wine*, and *ferrated wine* of wild cherry is, not that they are specifics in any one disease, but that these preparations can benefit, and have benefited patients suffering from *tuberculous consumption*, *chlorosis*, *anœmia*, *dyspepsia*, *debility* arising from various causes, *convalescence* from acute fevers, etc., etc.

They have, in addition, the merit of being very palatable, a point not always sufficiently regarded.

A medicine is none the less efficacious certainly because it has a pleasant taste. When we can *conciliate* the palate at the same time we are attacking a disease, we shall be quite as speedily successful in our efforts. Certainly, with children and fastidious invalids who have been compelled to *dose* largely, it is a merit to bring a medicine that shall be pleasant to take, and potential as a remedy.

